# Free thyroxine measured by chemiluminescence and equilibrium dialysis is frequently below the reference interval in known euthyroid dogs with nonthyroidal illness syndrome

**DOI:** 10.3389/fvets.2025.1657215

**Published:** 2025-10-10

**Authors:** Timothy A. Bolton, Christina L. Marino, Maxime G. Derré, George E. Moore, Julie Piccione, Malcolm L. Delovio

**Affiliations:** ^1^Department of Veterinary Clinical Sciences, College of Veterinary Medicine, Purdue University, West Lafayette, IN, United States; ^2^Small Animal Veterinary Specialists, Wyndmoor, PA, United States; ^3^College of Veterinary Medicine, Purdue University, West Lafayette, IN, United States; ^4^Department of Veterinary Administration, College of Veterinary Medicine, Purdue University, West Lafayette, IN, United States; ^5^Texas A&M Veterinary Medical Diagnostic Laboratory, College Station, TX, United States

**Keywords:** euthyroid sick syndrome, hypothyroidism, thyroid hormone assays, dog, endocrinology

## Abstract

**Introduction:**

Measuring serum free thyroxine (fT4) concentration is recommended to distinguish hypothyroidism from nonthyroidal illness syndrome (NTIS) in dogs having a serum total thyroxine (TT4) concentration below the reference interval (RI). Serum fT4 concentration can be measured by equilibrium dialysis (fT4ED) or chemiluminescent immunoassay (fT4CLIA), neither of which have been extensively evaluated in known euthyroid dogs with NTIS. The objectives of this study were to: (1) determine agreement between fT4ED and fT4CLIA in serum samples from established euthyroid dogs with NTIS, (2) evaluate how frequently each fT4 assay is within the RI when the serum TT4 concentration is within and below the RI, and (3) evaluate concordant and discordant fT4ED and fT4CLIA results in dogs with a serum TT4 concentration below the RI.

**Methods:**

This retrospective study used 116 banked serum samples from 38 known euthyroid dogs with NTIS. On each sample, fT4ED and fT4CLIA were measured, and agreement assessed by a Bland–Altman plot. Using the hospital admission serum TT4 concentration, dogs were stratified into two groups: TT4 within the RI (*n* = 16) and TT4 below the RI (*n* = 22). The frequency that each fT4 assay was within the RI was calculated in both groups. In dogs having a serum TT4 concentration below the RI, concordant and discordant fT4 results were evaluated.

**Results:**

Assay comparison showed agreement, with no significant systematic or proportional bias. When the serum TT4 concentration was within the RI, fT4ED and fT4CLIA were within the RI in 100% (95% CI: 79.4–100%) and 94% (95% CI: 69.8–99.8%) of dogs, respectively; however, when the serum TT4 concentration was below the RI, fT4ED and fT4CLIA were within the RI in 41% (95% CI: 20.7–63.6%) and 45% (95% CI: 24.4–67.8%) of dogs, respectively. Concordant fT4ED and fT4CLIA were present in 77% (95% CI: 54.6–92.2%) of dogs with a serum TT4 concentration below the RI.

**Discussion:**

The fT4ED and fT4CLIA were regularly below the RI in dogs with NTIS and a serum TT4 concentration below the RI, potentially resulting in a misdiagnosis of hypothyroidism. This raises concerns about the ability of both fT4 assays to distinguish hypothyroidism from NTIS in this subset of dogs.

## 1 Introduction

Thyroid function in the dog is evaluated by measuring serum thyroid hormone concentrations, particularly total thyroxine (TT4), free thyroxine (fT4), and thyroid-stimulating hormone (TSH). Up to 35% of euthyroid dogs with nonthyroidal illness syndrome (NTIS) demonstrate a decrease in serum TT4 concentration below the reference interval (RI), an alteration that frequently results in the incorrect diagnosis of hypothyroidism and unnecessary thyroxine (T4) supplementation in a euthyroid dog (1–3). Measuring serum fT4 concentration is recommended to distinguish NTIS from hypothyroidism in dogs with a low serum TT4 concentration since it more accurately reflects the functional status of the thyroid gland by quantifying the amount of T4 available to the tissues (4).

Equilibrium dialysis (ED) is considered the most accurate stand-alone test for the measurement of serum fT4 concentration since it separates the free fraction of T4 from serum binding proteins, protein binding inhibitors, and autoantibodies, such as T4 autoantibodies, that falsely increase the serum TT4 concentration, and is less affected by NTIS (1–3, 5–8). Despite these advantages, ED is technically demanding, time-consuming, and expensive, making it a cumbersome assay for use in veterinary laboratories processing large numbers of samples (6). Conversely, measurement of the serum fT4 concentration with a chemiluminescent immunoassay (CLIA) decreases the technical demand, time to results, and expense. Rather than dialysis to separate the free fraction of T4 from circulating proteins, CLIA employs a radioligand analog that has minimal interaction with these proteins (9). A veterinary-specific fT4 CLIA (fT4CLIA) purports to have a similar accuracy, but superior precision and turnaround time compared to fT4 measured by ED (fT4ED) (10). A recent study comparing fT4ED and fT4CLIA in dogs with NTIS revealed substantial agreement (Cohen’s kappa coefficient 0.63 [95% CI: 0.47–0.79]) between the 2 assays (11). In this study, greater than 90% of dogs had a serum fT4 concentration within the RI with both assays when the serum TT4 concentration was also within the RI; however, the fT4ED and fT4CLIA results were within the RI in only 43 and 12% of dogs, respectively, when the serum TT4 concentration was below the RI (11). These findings suggest that in dogs with NTIS and a low serum TT4 concentration, fT4ED is less reliable than previously reported at differentiating NTIS from hypothyroidism and that the fT4CLIA appears inferior to the fT4ED at such a differentiation (2, 3, 12). However, the inability to unambiguously exclude hypothyroid dogs when evaluating fT4 assays in dogs with NTIS is a limitation of the previous studies (2, 3, 11). The most recent study comparing fT4ED and fT4CLIA might have unintentionally included hypothyroid dogs and knowingly included dogs administered medications that can falsely lower the serum fT4 concentration, possibly contributing to the high percentage of serum fT4 concentrations below the RI (11). To address this limitation, a comparable study investigating these fT4 assays in established euthyroid dogs with NTIS was needed. Because euthyroid dogs with NTIS and serum TT4 and fT4 concentrations below the RI are frequently misdiagnosed and mistreated as hypothyroid in clinical practice, reassessing the capability of the fT4ED and fT4CLIA to distinguish NTIS from hypothyroidism in known euthyroid dogs with a serum TT4 concentration below the RI is clinically relevant.

The objectives of this study were three-fold: (1) to determine the agreement between fT4ED and fT4CLIA in serum samples from known euthyroid dogs with NTIS, (2) to evaluate how frequently each fT4 assay is within the RI when the serum TT4 concentration is within the RI and below the RI, and (3) to assess concordant and discordant fT4ED and fT4CLIA results in dogs with a serum TT4 concentration below the RI. Our first hypothesis was that the fT4 assays would agree and not be statistically different. Our second hypothesis was that each fT4 assay would have a result within the RI with high frequency and low frequency when the serum TT4 concentration was within and below the RI, respectively; however, since only euthyroid dogs were included in this study, the frequency of fT4 results within the RI when the serum TT4 concentration was below the RI would be higher than in a previous study (11).

## 2 Materials and methods

### 2.1 Current study

This was a retrospective study utilizing banked serum samples collected from client-owned dogs that participated in a prospective, observational study performed at the Virginia-Maryland College of Veterinary Medicine (12). Banked samples from the original study were included in the current study if serum of sufficient volume was available. The current study was exempt from institutional animal care and use committee review since only banked samples were used.

One hundred sixteen banked samples from 38 euthyroid dogs with NTIS had a sufficient volume of serum remaining to simultaneously measure fT4ED and fT4CLIA in the current study.

### 2.2 Original study

The original study received approval by the Virginia-Maryland College of Veterinary Medicine Institutional Animal Care and Use Committee, and informed owner consent was obtained prior to enrollment. Dogs were eligible for inclusion in the prospective, observational study to evaluate the alterations and time to resolution of thyroid function tests in dogs with NTIS if they suffered from acute illness (≤1-week duration) sufficiently severe to require hospitalization (12). Dogs fulfilling the inclusion criteria had a serum TT4 concentration measured at hospital admission. If the serum TT4 concentration was within the RI, no additional blood samples were obtained and the dog was excluded from the study. If the serum TT4 concentration was below the RI, further blood samples were obtained every 24 h after hospital admission until hospital discharge (acute phase) and at 2 weeks and 4 weeks following hospital discharge (recovery phase).

Exclusion criteria (no blood sample collected) included sighthounds, a history of chronic disease (prior disease diagnosis requiring ongoing treatment for management of clinical signs or current illness >1-week duration), and administration of a medication(s) known to affect thyroid function within 60 days of hospital admission. Excluded medications were acetylsalicylic acid, amiodarone, glucocorticoids, phenobarbital, sulfonamides, thyroid hormone replacement, toceranib, tricyclic antidepressants, trilostane, and zonisamide.

During the study, each blood sample collected was immediately placed into a siliconized glass vacutainer tube containing no anticoagulant and centrifuged at 3,000 g for 10 min. Serum was harvested, aliquoted into two separate polypropylene red top tubes (1 mL serum into one tube and the remaining serum into a second tube), frozen at −80 °C within 1 h of collection, and stored at −80 °C until analysis. As each dog completed the study, batch submission of serum samples (tubes containing 1 mL serum) for the measurement of serum concentrations of TT4, fT4, TSH, and total triiodothyronine was performed. All dogs analyzed in the study were deemed euthyroid based on the resolution of all serum thyroid hormone abnormalities found in both phases of illness, including low serum TT4, low serum fT4, and/or high serum TSH concentrations. Dogs excluded at the time of hospital admission due to a serum TT4 concentration within the RI were also deemed euthyroid.

One hundred banked samples from 22 euthyroid dogs with NTIS and a serum TT4 concentration below the RI at hospital admission (1–8 samples per dog obtained at 24 h to 2 week intervals) evaluated in the original study had a sufficient volume of serum remaining for use in the current study and 16 banked samples from 16 euthyroid dogs with NTIS (1 sample per dog) excluded from the original study because of a serum TT4 concentration within the RI at hospital admission had a sufficient volume of serum remaining for use in the current study.

### 2.3 Clinicopathologic analyses

#### 2.3.1 Serum fT4ED and fT4CLIA concentrations

One hundred sixteen banked serum samples were submitted to a single American Association of Veterinary Laboratory Diagnosticians (AAVLD) accredited laboratory (Texas A&M Veterinary Medical Diagnostic Laboratory (TVMDL), College Station, Texas) for concurrent measurement of serum fT4 concentration using fT4ED and fT4CLIA. Serum samples included in the present study were collected from January 2020 to December 2022 and were analyzed in January 2024. Because the aliquots used in the current study were stored at −80 °C until analysis, all samples only underwent a single-freeze–thaw cycle after they were batch shipped on dry ice to the TVMDL.

On each serum sample, simultaneous measurement of serum fT4 concentration by fT4ED (Vet Direct fT4 by ED, IVD Technologies, Santa Ana, California) and fT4CLIA (Immulite 2000 XPi, Veterinary fT4, Siemens, Erlangen, Germany) was performed. The fT4ED assay was performed in duplicate on each serum sample and an average was reported.

The fT4ED assay is a commercially available kit validated in accordance with AAVLD standards through onboarding verification, ongoing proficiency testing, and daily control charting. The assay has reported limits of detection and quantification of <1.29 pmol/L and 2.57 pmol/L, respectively, average mean accuracy of 94.6%, analytical range of 2.57 to 164.76 pmol/L, intra-assay variations of 14.3, 11.2, and 6.5% at mean serum fT4 concentrations of 10.30 pmol/L, 23.17 pmol/L, and 55.35 pmol/L, respectively, and inter-assay variations of 16.2, 10.5, and 8.4% at mean serum fT4 concentrations of 10.30 pmol/L, 21.88 pmol/L, and 50.20 pmol/L, respectively (13). The assay RI of 7.20 to 36.70 pmol/L was determined by TVMDL in accordance with the American Society for Clinical Pathology (ASVCP) Quality Assurance and Laboratory Standards guidelines (14).

The fT4CLIA assay is a commercially available kit whose accuracy has been evaluated in both euthyroid and hypothyroid dogs (15). It has been validated in accordance with AAVLD standards through onboarding verification, ongoing proficiency testing, and daily control charting. The assay has reported limits of detection and quantification of 1.29 pmol/L and 3.86 pmol/L, respectively, analytical range of 3.86 to 77.20 pmol/L, intra-assay variations of 10.8, 5.9, and 3.5% at mean serum fT4 concentrations of 7.59 pmol/L, 24.20 pmol/L, and 75.56 pmol/L, respectively, and inter-assay variations of 11.9, 6.5, and 4.3% at similar mean serum fT4 concentrations as listed for the intra-assay variation (16). The assay RI of 7.10 to 32.70 pmol/L was determined by TVMDL in accordance with the ASVCP Quality Assurance and Laboratory Standards guidelines (14).

#### 2.3.2 Serum TT4 concentration

The serum TT4 concentration measured on each hospital admission sample in the original study was used in the present study to separate the 38 dogs into two groups based on whether the serum TT4 concentration was within or below the RI. In the original study, the serum TT4 concentration was measured with a CLIA (Immulite 2000 XPi, Siemens, Erlangen, Germany) at the Virginia-Maryland College of Veterinary Medicine clinical pathology laboratory with a RI of 12.8 to 36.3 nmol/L and lower limit of quantification of 6.4 nmol/L (17).

### 2.4 Statistical analysis

#### 2.4.1 Agreement between fT4ED and fT4CLIA

The fT4ED and fT4CLIA results from all 116 banked serum samples were included in objective 1. Agreement between serum concentrations of fT4ED and fT4CLIA was assessed using a Bland–Altman plot and the calculation of systematic and proportional bias. Systematic bias was measured as the arithmetic mean difference, with its 95% limits of agreement (±1.96 standard deviation), between methods of the concentrations of the same sample. Proportional bias was measured as the slope, with its 95% confidence interval, of the regression equation of method differences plotted against the mean of the two methods. All calculations were performed using commercial statistical software (MedCalc Statistical Software, Ostend, Belgium, 2013). *p*-values < 0.05 were significant.

For statistical purposes, when the serum fT4 concentration obtained by ED or CLIA was below the limits of quantification, a value of 2.56 pmol/L or 3.85 pmol/L was assigned, respectively.

#### 2.4.2 Frequency of fT4ED and fT4CLIA within the RI

The fT4ED and fT4CLIA results from 38 banked serum samples, representing each dog’s sample at hospital admission, were included in objective 2. The serum concentrations of fT4ED, fT4CLIA, and TT4 on each serum sample were documented as below, within, or above the RI. The frequency of fT4 results within the RI for each fT4 assay was calculated when the serum TT4 concentration was within and below the RI.

#### 2.4.3 Concordance and discordance of fT4ED and fT4CLIA

For each of the 22 dogs having a serum TT4 concentration below the RI at hospital admission, fT4ED and fT4CLIA results from only the hospital admission sample were included in objective 3. When fT4ED and fT4CLIA results were similar (both within the RI or below the RI), they were deemed concordant, and when fT4ED and fT4CLIA results were different (one within the RI and one below the RI), they were deemed discordant. Magnitude of disparity from the RI and disease diagnoses in the dogs were evaluated.

## 3 Results

### 3.1 Agreement between fT4ED and fT4CLIA

**Figure 1:**
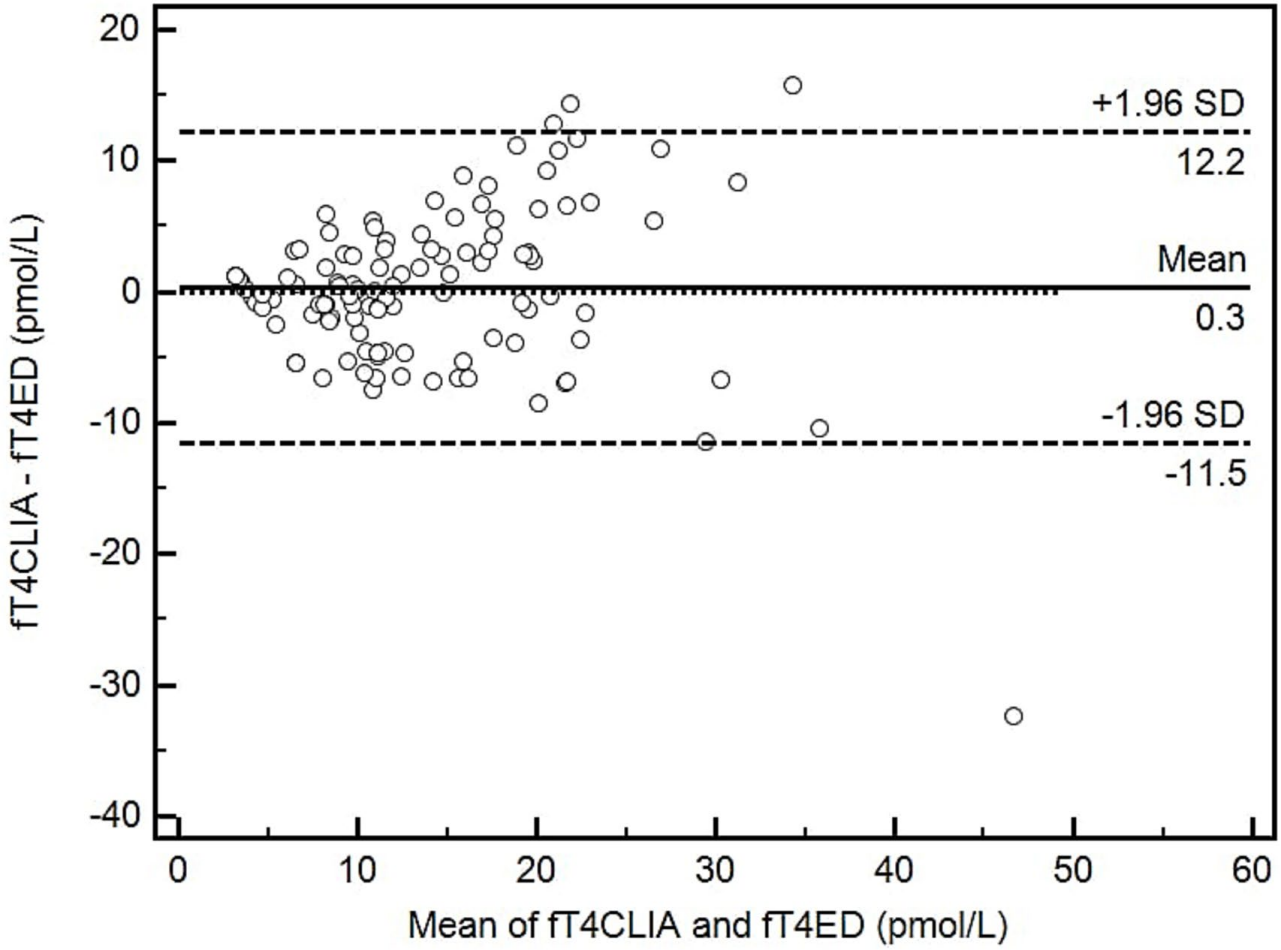
Bland–Altman plot depicting the comparison of means to differences between 2 methods of serum free thyroxine measurement (fT4ED and fT4CLIA). Open circles represent the 116 serum samples. The solid line represents the arithmetic mean difference; dashed lines represent the 95% limits of agreement. The dotted line would indicate perfect agreement between methods. fT4CLIA, free thyroxine by chemiluminescent immunoassay; fT4ED, free thyroxine by equilibrium dialysis.

No significant systematic bias (arithmetic mean difference, 0.3 pmol/L; 95% limits of agreement, −11.5 to 12.2 pmol/L; *p* = 0.54) or proportional bias (slope, −0.03; 95% CI, −0.18 to 0.12; *p* = 0.70) was identified when comparing fT4ED to fT4CLIA (Figure 1).

### 3.2 Frequency of fT4ED and fT4CLIA within the RI

Upon hospital admission, 16 dogs had a serum TT4 concentration within the RI and 22 dogs had a serum TT4 concentration below the RI. In dogs with a serum TT4 concentration within the RI, fT4ED and fT4CLIA were within the RI in 100% (16/16; 95% CI: 79.4–100%) and 94% (15/16; 95% CI: 69.8–99.8%) of dogs, respectively. One dog had a fT4CLIA result above the RI while the corresponding fT4ED result was within the RI. In dogs with a serum TT4 concentration below the RI, fT4ED and fT4CLIA were within the RI in 41% (9/22; 95% CI: 20.7–63.6%) and 45% (10/22; 95% CI: 24.4–67.8%) of dogs, respectively. Conversely, fT4ED and fT4CLIA were below the RI in 59% (13/22; 95% CI: 36.4–79.3%) and 55% (12/22; 95% CI: 32.2–75.6%) of dogs, respectively, with a serum TT4 concentration below the RI.

### 3.3 Concordance and discordance of fT4ED and fT4CLIA

In the 22 dogs with a serum TT4 concentration below the RI at hospital admission, concordant results were present in 77% (17/22; 95% CI: 54.6–92.2%) of dogs. Both assays were below the RI in 10 dogs (dogs 1–10; Table 1) and within the RI in 7 dogs (dogs 16–22; Table 1). Of the 10 dogs with serum fT4 concentrations below the RI by both assays, the fT4ED was undetectable in 2 dogs (dogs 1 and 2; Table 1), ≥1.70 pmol/L below the RI in 7 dogs (dogs 3–9; Table 1), and 1.00 pmol/L below the RI in 1 dog (dog 10; Table 1). In the same 10 dogs, the fT4CLIA was undetectable in 5 dogs (dogs 1–5; Table 1), >2.10 pmol/L below the RI in 3 dogs (dogs 6–8; Table 1), and within 0.45 pmol/L of the low end of the RI in 2 dogs (dogs 9 and 10; Table 1). Discordant results were present in 23% (5/22; 95% CI: 7.8–45.4%) of dogs. The fT4ED was below the RI and the fT4CLIA was within the RI in 3 dogs (dogs 11–13; Table 1), whereas the fT4ED was within the RI and the fT4CLIA was below the RI in 2 dogs (dogs 14 and 15; Table 1). Regardless of fT4 assay, all results below the RI among the discordant dogs were >1.90 pmol/L below the RI (dogs 11–15; Table 1). The disease diagnoses for the concordant and discordant fT4ED and fT4CLIA results are presented in Table 1.

**Table 1:** Concordant and discordant fT4ED and fT4CLIA results in dogs with a low serum total thyroxine concentration.

		RI: 7.20–36.70 pmol/L	RI: 7.10–32.70 pmol/L	
Dog	Disease diagnosis	fT4ED	fT4CLIA	Concordant/discordant
1	Gastrointestinal obstruction	<2.57 (L)	<3.86 (L)	Concordant
2	Acute gastroenteritis	<2.57 (L)	<3.86 (L)	Concordant
3	Bacterial bronchopneumonia	3.61 (L)	<3.86 (L)	Concordant
4	Parvoviral enteritis	4.29 (L)	<3.86 (L)	Concordant
5	Pyometra	4.70 (L)	<3.86 (L)	Concordant
6	Bacterial bronchopneumonia	4.80 (L)	4.62 (L)	Concordant
7	Pyometra	5.26 (L)	4.07 (L)	Concordant
8	Acute gastroenteritis	5.49 (L)	4.98 (L)	Concordant
9	Acute gastroenteritis	5.50 (L)	6.67 (L)	Concordant
10	Bacterial prostatitis/epididymitis	6.20 (L)	6.81 (L)	Concordant
11	Acute gastroenteritis	4.84 (L)	7.99 (N)	Discordant
12	Traumatic pneumothorax	5.11 (L)	8.38 (N)	Discordant
13	Gastrointestinal obstruction	5.28 (L)	11.20 (N)	Discordant
14	Gastrointestinal obstruction	9.31 (N)	<3.86 (L)	Discordant
15	Pyothorax	11.26 (N)	4.74 (L)	Discordant
16	Gastrointestinal obstruction	9.48 (N)	7.50 (N)	Concordant
17	Shock of unknown etiology	11.70 (N)	8.56 (N)	Concordant
18	Acute gastroenteritis	13.32 (N)	16.10 (N)	Concordant
19	Drug-induced hepatopathy	13.39 (N)	8.76 (N)	Concordant
20	Lung lobe torsion	19.30 (N)	15.80 (N)	Concordant
21	Gallbladder mucocele	24.30 (N)	15.80 (N)	Concordant
22	Gastrointestinal obstruction	25.01 (N)	18.10 (N)	Concordant

fT4ED, free thyroxine by equilibrium dialysis; fT4CLIA, free thyroxine by chemiluminescent immunoassay.

(L), below the reference interval; (N), within the reference interval; RI, reference interval.

## 4 Discussion

A serum fT4 concentration measured by ED has been considered the most accurate stand-alone test for distinguishing NTIS from hypothyroidism (1–3); however, prior studies present conflicting data on the frequency that fT4ED and fT4CLIA are within the RI in sick dogs with a serum TT4 concentration below the RI (2, 3, 11, 12). In our cohort of established euthyroid dogs with NTIS, the fT4ED and fT4CLIA demonstrated agreement and had no significant bias; nevertheless, the small systematic bias of 0.3 pmol/L and wide 95% limits of agreement could influence clinical decision making, especially when assay results are near the low end of the RI. While >90% of dogs with a serum TT4 concentration within the RI also had a serum fT4 concentration within the RI by either assay, <50% of dogs with a serum TT4 concentration below the RI had a serum fT4 concentration within the RI by either assay. Most of the dogs with a fT4ED, fT4CLIA, or both below the RI had an inflammatory disease that likely affected assay results. In dogs with NTIS caused by an acute, resolvable disease, a clinician should exercise caution when interpreting concurrent serum TT4 and fT4ED or fT4CLIA concentrations below the RI because it could result in a misdiagnosis of hypothyroidism.

When comparing fT4ED and fT4CLIA, neither significant systematic nor proportional bias was present; however, this does not mean these assays are interchangeable. The small systematic bias of 0.3 pmol/L means that, on average, fT4ED measures 0.3 pmol/L lower than fT4CLIA. A visual inspection of the Bland–Altman plot demonstrates larger differences between fT4ED and fT4CLIA at serum fT4 concentrations >10 pmol/L and smaller differences between fT4ED and fT4CLIA at serum fT4 concentrations <10 pmol/L. Furthermore, the 95% limits of agreement are wide in order to include the large differences between fT4ED and fT4CLIA at higher serum fT4 concentrations. Luckily, large differences between fT4ED and fT4CLIA at higher serum fT4 concentrations does not impact clinical decision making when attempting to exclude hypothyroidism; nonetheless, the small and statistically insignificant systematic bias near the low end of the RI may be of sufficient magnitude to falsely lower the serum fT4 concentration below the RI. Consequently, a serum fT4 concentration just below the low end of the RI measured by either assay should be interpreted with caution and in light of the clinical picture.

In euthyroid dogs with NTIS and a serum TT4 concentration below the RI, fT4ED and fT4CLIA were also below the RI in 59 and 55% of dogs, respectively. While these findings highlight the limited ability of these assays to reliably exclude hypothyroidism in this cohort, it is notable that almost 50% of the dogs had a serum fT4 concentration within the RI by either assay. In such cases, a serum fT4 concentration might still provide useful clinical guidance on the true thyroid status of the dog, particularly when gold-standard testing (i.e., TSH stimulation or thyroid scintigraphy) is unavailable. However, a recent study reported that roughly 50% of dogs in first opinion practices receiving levothyroxine were likely being administered this medication unnecessarily, supporting the notion that hypothyroidism is overly and incorrectly diagnosed (18). Reasons for this include inherent limitations to the thyroid function tests and the influence of external factors on such tests, such as NTIS and medications (18). Unnecessary thyroid hormone supplementation subjects dogs to repeat and lifelong blood sampling for monitoring purposes and can delay the diagnosis of the true underlying disease. Given the frequency of serum TT4 and fT4 concentrations below the RI in euthyroid dogs with NTIS, clinicians should only perform thyroid function testing in those dogs with a high clinical suspicion for the disease.

Serum fT4 assays were developed for more accurate measurement of T4, either via separating the free fraction of T4 from serum proteins (ED) or using a radioligand analog that binds directly to T4 without interference from serum proteins (CLIA). Having intraassay variations of 14.3 and 10.8% for the fT4ED and fT4CLIA, respectively, when serum fT4 concentrations are near the low end of the RI, assay results <1.03 pmol/L below the low end of the RI for fT4ED and <0.77 pmol/L below the low end of the RI for fT4CLIA would fall within the published intra-assay variation for each assay. If repeated, the serum fT4 concentration may fall within the RI. Since only 1 dog (dog 10; Table 1) with a fT4ED result and 2 dogs (dogs 9 and 10; Table 1) with a fT4CLIA result fell within the intra-assay variation for each assay, it appears unlikely that serum fT4 concentrations below the RI by either assay are a result of inherent assay imprecision and are more likely due to factors in each dog related to the severity and specific illnesses present. Interestingly, most dogs having a serum fT4 concentration below the RI by one or both assays were diagnosed with an inflammatory disease (Table 1). In an inflammatory disease, acute phase proteins (APPs) increase by more than 25% in the serum and can consequently lower T4 measurement (19, 20). Furthermore, it has been demonstrated that fT4ED and fT4CLIA have decreasing concentrations as the severity of the illness increases, possibly due to the presence of serum proteins that interfere with the fT4 assays, such as APPs (2, 11). Because all dogs in the present study with a serum TT4 concentration below the RI were hospitalized, indicating, at minimum, a moderate severity of illness based on a disease severity stratification published in a previous study (2), it is plausible that APPs interfered with the ability of both assays to accurately measure T4 in dogs with a fT4ED, fT4CLIA, or both below the RI (dogs 1–15; Table 1).

In this study, 45% of dogs with NTIS and a serum TT4 concentration less than the RI had a fT4CLIA result within the RI compared to just 12% in a previous study (11). While the fT4CLIA performed better in the present study, both results are unacceptably poor in clinical practice when used to rule out hypothyroidism in this cohort of dogs. The findings of this study corroborate those of previous studies whereby the fT4CLIA (same methodology) is of little additional diagnostic value both in dogs with NTIS and a serum TT4 concentration below the RI (11), and in dogs being investigated for potential hypothyroidism (21). While still poor, the superior performance of the fT4CLIA in the present study could be, in part, due to the inclusion of only known euthyroid dogs and exclusion of dogs administered medications that are known to lower the serum fT4 concentration (11, 22). The severity and specific illnesses present between the 2 studies are likely different and can explain the discordant finding; however, a direct comparison between studies is difficult due to the lack of data on these variables in the earlier study (11). Of note, while the fT4CLIA used in these studies was the same, the laboratory performing the analysis in the present study internally validated the fT4CLIA assay and generated a laboratory-specific RI, none of which was reported in the prior study (11).

Earlier studies evaluating the fT4ED assay in dogs with NTIS discovered that greater than 80% of dogs had a serum fT4 concentration within the RI (1–3). This high percentage created a favorable opinion of the fT4ED assay, perpetuating the assertion that it is the most accurate stand-alone diagnostic test for differentiating NTIS and hypothyroidism; however, it is important to understand that this high percentage represents an aggregate of dogs with serum TT4 concentrations within and below the RI without separating out the latter group of dogs (1–3). Since measurement of the serum fT4 concentration is usually reserved for the differentiation of NTIS and hypothyroidism in dogs with a serum TT4 concentration below the RI, evaluation of the fT4ED in this cohort is more clinically relevant. In 4 previous studies evaluating dogs with NTIS using fT4ED, the serum fT4 concentration was within the RI when the serum TT4 concentration was below the RI in 43% (11), 55% (2), 86% (12), and 97% (3) of dogs. Notably, in the previous study (12) using the same serum samples, 86% of dogs with a low serum TT4 concentration had a serum fT4 concentration by ED within the RI, compared to only 41% in the current study. All prior studies (2, 3, 11, 12) used the same ED assay (Antech Diagnostics, Irvine, CA), whereas the current study employed a different ED assay (IVD Technologies, Santa Ana, CA). This decision introduced methodology variation; however, it was made to ensure consistency in sample handling and reduce pre-analytical variation. Given that the serum fT4 concentration is stable in human serum samples stored frozen for a long period of time, sample degradation is unlikely to explain the discrepancy (23). Instead, it is possible that the ED assay used in the current study underperformed compared to the previously used assay. Nonetheless, the clinical consequence of all these findings is that the fT4ED assay can correctly diagnose NTIS and exclude hypothyroidism in 41–97% of dogs with illness. Taking all of this into consideration, the reliability of fT4ED at differentiating NTIS from hypothyroidism in dogs with a serum TT4 concentration below the RI, the clinically relevant group, is questionable.

Overall, ED assay results are extremely variable, with up to 90% variability in humans when the serum fT4 concentration is at the lower end of the RI (24). Because of this, human laboratories have key standardized components when performing the ED assay, particularly using a buffer that mimics the ionic environment of the serum, ensuring a constant temperature of the dialysate, using an undiluted serum sample, and obtaining a standard serum pH prior to performing the ED assay (25). Despite these standardizations, variation in the serum fT4 concentration of as much as 50% is expected when the serum sample is analyzed at a different reference laboratory (26). As of right now in veterinary medicine, standardization does not occur; however, using an AAVLD accredited laboratory with robust quality guidelines, validation requirements, standard operating procedures, and duplicate assay runs limits result variation. Nonetheless, analyzing serum fT4 concentrations at different veterinary reference laboratories can still result in variation of up to 25% in the ED assay result (27). The inferior performance of the fT4ED in the present study as compared to prior studies (2, 3, 11, 12) could be because it was a different ED assay run at a different laboratory or the result of assay performance in the face of illness. While the dogs with a serum TT4 concentration below the RI in the present study were not stratified based on severity of disease, all dogs required hospitalization for inclusion in the study from which these samples originated, meaning they had, at minimum, illness of moderate severity (2). Because increasing severity of illness results in an incrementally lower serum fT4 concentration, the inferior performance of the fT4ED in this study and others (2, 11) could be due to the severity or even type of illnesses present. Thus, the ability of the fT4ED assay to rule out hypothyroidism in an ill dog having a serum TT4 concentration below the RI (likely a dog with moderate to severe illness) is low and clinically unacceptable. To limit some of the variation seen with the fT4ED assay, however, the submission of all serum samples for a single patient to the same reference laboratory is recommended.

Our study had some limitations. First, serum samples were stored at −80 °C for up to 4 years prior to analysis making sample degradation a possibility. Although no data is available on the effect of long-term storage at −80 °C on serum thyroid hormone concentrations in dogs with NTIS, the serum fT4 concentration is stable and can therefore be reliably analyzed in human serum samples stored for up to 23 years at −25 °C (23). Additionally, all serum samples were kept frozen until analysis so only endured one freeze–thaw cycle to limit potential degradation.

Second, a different ED assay was used in this study compared to 4 previous studies (2, 3, 11, 12). This decision was made to minimize pre-analytical variability by selecting an AAVLD-accredited laboratory able to perform both the fT4ED and fT4CLIA assays concurrently on the same serum aliquot with only a single freeze–thaw cycle. While this approach improved consistency in sample handling, it required the use of a different ED assay kit, as the assay used in the prior studies is not readily available to most laboratories. The decrease in assay performance in this study compared to the previous study (12) using these same serum samples may be secondary to the ED assay used; however, this reflects the reality of clinical practice where clinicians typically cannot choose the ED assay used when submitting serum samples. Because fT4ED results in the original study (12) were obtained at a different time and under different conditions, we did not include them in this study, as doing so would have introduced additional confounding variables.

Third, our study investigated dogs with acute resolvable illness affecting limited body systems and for which stratification based on disease severity was not performed due to the criteria under which these samples were obtained (12), unlike previous studies where dogs with both acute and chronic illness affecting more than 10 body systems and stratified based on disease severity were evaluated (1–3, 11). While this might limit the applicability of our findings to all dogs with NTIS, the study design guaranteed that only known euthyroid dogs were included.

Fourth, despite a large number of serum samples available for the method comparison portion of our study (objective 1), each sample did not represent a unique dog and the range of serum fT4 concentrations associated with these samples might be considered narrow. Unfortunately, because our study was retrospective, these limitations were unavoidable; however, unlike correlation or agreement coefficients, Bland–Altman analysis is preferred for assessing agreement between two methods (28), and serum samples from the same dog represented different time points within that particular dog’s illness with each sample collected at least 24 h apart. Alternatively, assessing agreement between ED and CLIA with one sample per dog would have substantially decreased the number of samples and range of serum fT4 concentrations analyzed, potentially resulting in type II error. Therefore, a decision was made to pursue the current analysis.

Finally, the number of dogs used to evaluate the frequency that each fT4 assay was within the RI and subsequently diagnose the dog with NTIS and not hypothyroidism was small. Additional studies with a larger group of known euthyroid dogs with NTIS with more diseases is warranted.

In conclusion, while fT4ED and fT4CLIA measured with these specific assays in this cohort of dogs were not statistically different, careful interpretation of a serum fT4 concentration just below the lower limit of the RI is recommended. In dogs with a serum TT4 concentration below the RI, the serum fT4 concentration was within the RI in less than 50% of dogs, demonstrating limited reliability for ruling out hypothyroidism in dogs with NTIS. However, both assays were capable of appropriately ruling out hypothyroidism in almost half of the dogs, suggesting that when using these particular assays, neither needs to be preferentially selected over the other. When both serum TT4 and fT4 concentrations are below the RI, clinical context becomes crucial, especially if gold-standard testing is not feasible or available. In these cases, rechecking thyroid function tests after resolution of the illness may provide diagnostic clarity.

## Data Availability

The original contributions presented in the study are included in the article/supplementary material, further inquiries can be directed to the corresponding author.
